# Probiotic *Lactobacillus* spp. improves *Drosophila* memory by increasing lactate dehydrogenase levels in the brain mushroom body neurons

**DOI:** 10.1080/19490976.2024.2316533

**Published:** 2024-02-19

**Authors:** Shuk-Man Ho, Wan-Hua Tsai, Chih-Ho Lai, Meng-Hsuan Chiang, Wang-Po Lee, Hui-Yu Wu, Pei-Yi Bai, Tony Wu, Chia-Lin Wu

**Affiliations:** a Department of Biochemistry, Department of Microbiology and Immunology, Graduate Institute of Biomedical Sciences, Department of Biomedical Sciences, College of Medicine, Chang Gung University, Taoyuan, Taiwan; b Research and Development Department, GenMont Biotech Incorporation, Tainan, Taiwan; c Molecular Infectious Disease Research Center, Department of Pediatrics, Linkou Chang Gung Memorial Hospital, Taoyuan, Taiwan; d Department of Medical Research, School of Medicine, China Medical University and Hospital, Taichung, Taiwan; e Department of Nursing, Asia University, Taichung, Taiwan; f Research Center for Emerging Viral Infections, Chang Gung University, Taoyuan, Taiwan; g Department of Neurology, New Taipei Municipal TuCheng Hospital, Chang Gung Memorial Hospital, New Taipei City, Taiwan; h Brain Research Center, National Tsing Hua University, Hsinchu, Taiwan

**Keywords:** Probiotics, *Lactobacillus*, Brain, Olfactory memory, Lactate dehydrogenase, *Drosophila melanogaster*

## Abstract

Probiotics are live microorganisms that offer potential benefits to their hosts and can occasionally influence behavioral responses. However, the detailed mechanisms by which probiotics affect the behavior of their hosts and the underlying biogenic effects remain unclear. Lactic acid bacteria, specifically *Lactobacillus* spp. are known probiotics. *Drosophila melanogaster*, commonly known as the fruit fly, is a well-established model organism for investigating the interaction between the host and gut microbiota in translational research. Herein, we showed that 5-day administration of *Lactobacillus acidophilus* (termed GMNL-185) or *Lacticaseibacillus rhamnosus* (termed GMNL-680) enhances olfactory-associative memory in *Drosophila*. Moreover, a combined diet of GMNL-185 and GMNL-680 demonstrated synergistic effects on memory functions. Live brain imaging revealed a significant increase in calcium responses to the training odor in the mushroom body β and γ lobes of flies that underwent mixed feeding with GMNL-185 and GMNL-680. Quantitative reverse transcription polymerase chain reaction (qRT-PCR) and whole-mount brain immunohistochemistry revealed significant upregulation of lactate dehydrogenase (LDH) expression in the fly brain following the mixed feeding. Notably, the genetic knockdown of *Ldh* in neurons, specifically in mushroom body, ameliorated the beneficial effects of mixed feeding with GMNL-185 and GMNL-680 on memory improvement. Altogether, our results demonstrate that supplementation with *L. acidophilus* and *L. rhamnosus* enhances memory functions in flies by increasing brain LDH levels.

## Introduction

A well-balanced gut microbiota is important for human health, and altering the composition of the gut microbiota could potentially impact the physiological responses of the host, leading to neural and behavioral changes.^1–3^ Gut microbiome is influenced by the dietary preferences and innate immune system of the host.^4,5^ Gut microbes can produce and utilize various neurotransmitters, including norepinephrine, dopamine, serotonin, and gamma-aminobutyric acid (GABA),^6^ which affect the physiological responses of the hosts.^7^ Accumulated evidence suggests that the gut microbiome is intricately involved in virtually all aspects of nutrient metabolism within the host; however, the precise interactions between the gut microbiota and host physiology remain poorly understood.^8,9^ Probiotics are either single or combined live beneficial microorganisms, such as bacteria or yeast, that naturally inhabit the human body. Probiotic consumption is associated with several benefits, and it has been reported that probiotics could alleviate the symptoms of lactose intolerance, promote intestinal health, and reduce the risk of the development of various diseases.^10–13^

Previous studies investigating the benefits of the gut microbiota on host health predominantly employed mammalian models. Numerous studies have revealed the diversity of gut microbes in *Drosophila melanogaster* (*D. melanogaster*) and showed that *Acetobacter* and *Lactobacillus* are the two major species in the *Drosophila* gut.^14–16^ The fruit fly *D. melanogaster* has gained traction as an ideal model organism to explore host-gut microbe interactions in the realms of innate immunity, behavior, and neurological disorders.^15,17,18^ Owing to its intricate behavior and the availability of an array of genetic tools for tissue-specific gene manipulation, *Drosophila* permits a comprehensive examination of the underlying mechanisms behind both innate and learned behaviors. One widely utilized behavioral paradigm for evaluating *Drosophila* olfactory associative memory over the past four decades is the Pavlovian conditioning. In this paradigm, flies receive aversive electric foot shocks (unconditioned stimulus, US) in conjunction with exposure to specific odors (conditioned stimulus, CS+), followed by exposure to another odor (CS-) without electric foot shocks. Subsequently, flies can learn to avoid the odor associated with electric shocks, and this memory gradually diminishes over the initial 7 hours post-training, while it remains discernible 24 hours later.^19^ Olfactory memory formation in *Drosophila* is dependent on olfactory learning and memory center, the mushroom body.^20–26^ Mushroom bodies are paired neuropils composed of approximately 2000 neurons called Kenyon cells in each brain hemisphere.^27^ Mushroom body neurons in the *Drosophila* brain can be classified into three subsets, including γ, αβ, and α′β′. The axons of these neurons can be further divided into two vertical α and α′ lobes, and three horizontal lobes, including γ, β, and β′ lobes.^28,29^

Lactate dehydrogenase (LDH) serves as the rate-limiting enzyme in the conversion of lactate to pyruvate.^30^ This conversion, coupled with the interchange between oxidized and reduced forms of nicotinamide adenine dinucleotide (NAD), plays a role in maintaining carbohydrate metabolism equilibrium.^30^ LDH is distributed across various cell types, including muscle tissue, red blood cells, and organs, such as the liver and kidneys.^31^ It catalyzes the reversible conversion of lactate to pyruvate and vice versa during cellular metabolism.^30^ In *Drosophila*, glucose or trehalose catabolism yields pyruvate, which can be further oxidized by the pyruvate dehydrogenase (PDH) to form acetyl coenzyme A (acetyl-CoA).^32^ During aerobic conditions, acetyl-CoA is oxidized into carbon dioxide via the tricarboxylic acid (TCA) cycle within the mitochondrial matrix.^33^ During anaerobic glycolysis, pyruvate is reduced to lactate via LDH.^34^ In *D. melanogaster*, the *ImpL3/Ldh* gene encodes a single LDH isoform,^35^ which is expressed in both neurons and glia.^36,37^ It has been shown that *Drosophila* LDH plays a critical role in flight muscle development, maintenance of muscle integrity, and energy homeostasis.^38–42^

Herein, we identified two bacterial strains, *Lactobacillus acidophilus* and *Lacticaseibacillus rhamnosus*, referred to as GMNL-185 and GMNL-680, respectively that exhibited potent beneficial effects on olfactory memory in *Drosophila*. A 5-day ingestion of either GMNL-185 or GMNL-680 enhanced the memory functions in flies while mixed GMNL-185 and GMNL-680 feeding exhibited synergistic effects. Lived brain imaging revealed significantly enhanced neuronal responses to training odor in mushroom body β and γ lobes following GMNL-185 and GMNL-680 mixed feeding. Interestingly, we found that LDH expression was upregulated in the brains of flies fed a mixture of GMNL-185 and GMNL-680, and silencing the *Ldh* gene in neurons, specifically in the mushroom body, diminished the enhanced memory performance after the GMNL-185 and GMNL-680 mixed feeding. Thus, our study suggests that the probiotic GMNL-185 and GMNL-680 enhance brain LDH expression, thereby, contributing to the elevated mushroom body neuronal responses toward training odors and improved olfactory memory in *Drosophila*.

## Results

### Memory improvements following GMNL-185 and GMNL-680 feeding in *Drosophila*

**Figure 1.**
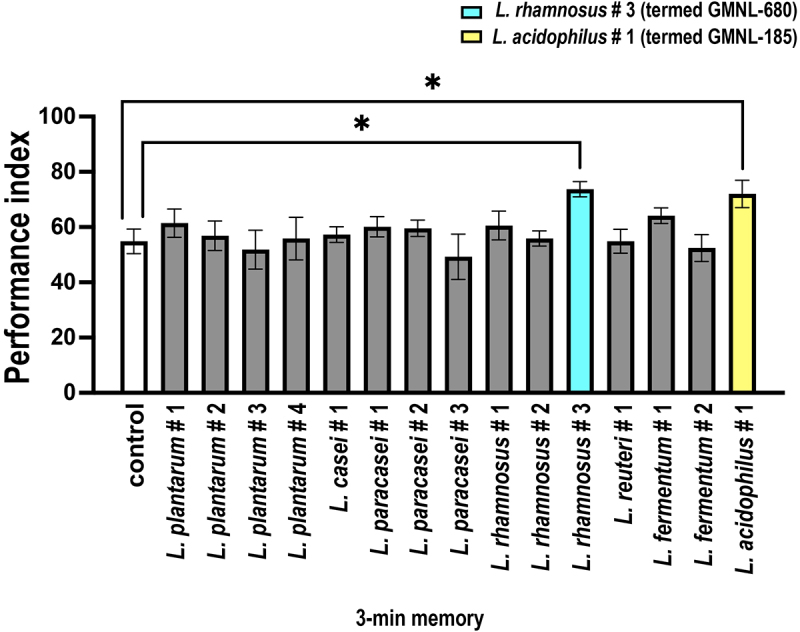
Flies fed with *Lactobacillus* spp. GMNL-680 or GMNL-185 exhibit enhanced 3-minute olfactory memory.

Several studies have shown the significance of gut microbiota in influencing interactions within the gut-brain axis^43,44^ and the impact of probiotic bacteria on cognitive functions. *Lactobacillus* spp. are considered potential probiotics.^45–48^ Herein, we investigated the effects of various *Lactobacillus* spp. on cognitive functions in *D. melanogaster*. We analyzed 15 different *Lactobacillus* strains, including 4 strains of *Lactiplantibacillus plantarum*, 1 strain of *Lacticaseibacillus casei*, 3 strains of *Lacticaseibacillus paracasei*, 3 strains of *Lacticaseibacillus rhamnosus*, 1 strain of *Limosilactobacillus reuteri*, 2 strains of *Limosilactobacillus fermentum*, and 1 strain of *Lactobacillus acidophilus*. These strains were obtained from the Probiotic Bank (GenMont Biotech, Tainan, Taiwan). Wild-type Canton-S flies were reared and fed different *Lactobacillus* strains for 5 days before performing the olfactory memory assays. Wild-type flies reared on food without *Lactobacillus* strains were used as controls (Figure 1). Results showed that no significant differences in the 3-minute memory among the 13 *Lactobacillus* strains fed flies compared to the control flies. However, flies subjected to GMNL-185 or GMNL-680 feeding exhibited a significant increase in the 3-minute memory compared to the control group (Figure 1).

Wild-type flies were individually reared on food containing different *Lactobacillus* spp. for five days, whereas the control group was reared on food without *Lactobacillus* spp. After five days, the flies were transferred to a T-maze for olfactory memory assays. During the assay, the flies were exposed to a CS+ odor (OCT or MCH) paired with 12 instances of 60-volt electric shock within 1 minute, and their 3-minute memory was tested immediately after the training. Notably, only flies that were fed *Lactobacillus* spp. *L. rhamnosus* #3 (termed GMNL-680) or *L. acidophilus* #1 (termed GMNL-185) showed a significant increase in the 3-minute memory. Each bar represents the mean ± SEM (*N* = 7); *p < 0.05, analyzed using the Student’s *t*-test.

### Live GMNL-185 and GMNL-680 strains in the *Drosophila* digestive tract after feeding

**Figure 2.**
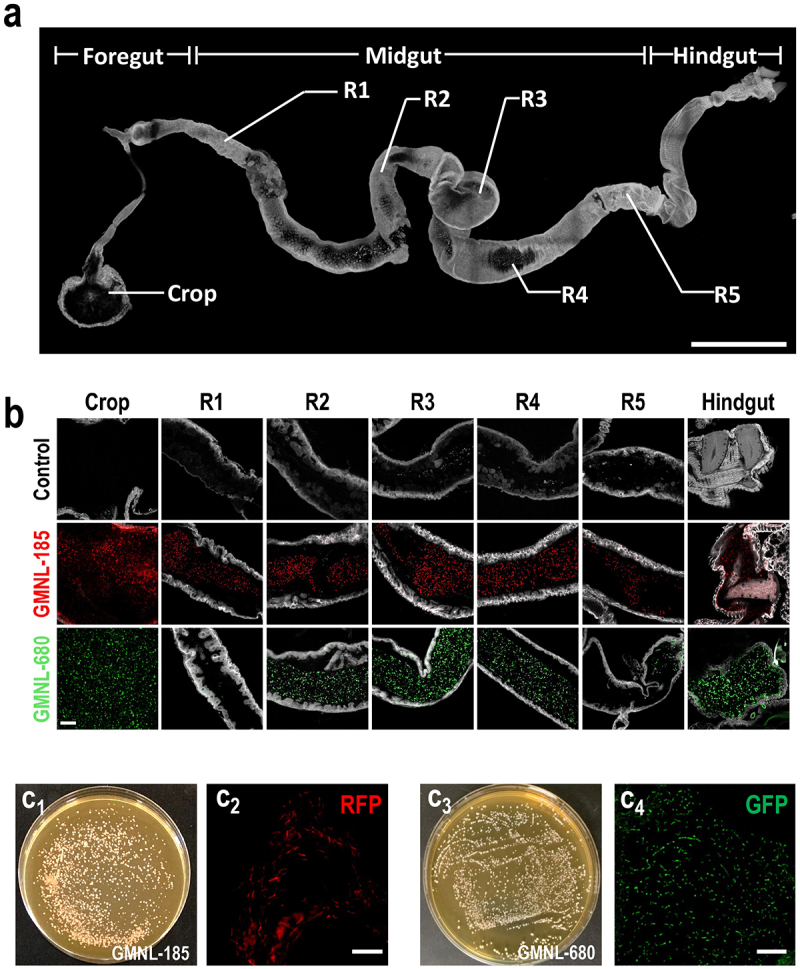
Live GMNL-185 and GMNL-680 strains in the fly digestive tracts after five days of feeding.

The digestive tract of adult flies can be anatomically divided into three regions, the foregut (crop), midgut (R1-R5), and hindgut^18,49^ (Figure 2a). We investigated the abundance of GMNL-185 and GMNL-680 within the digestive tract of flies following a 5-day *Lactobacillus* spp. feeding. Flies were raised in a medium containing RFP-tagged GMNL-185 or GFP-tagged GMNL-680 for 5 days, and the digestive tracts were isolated and imaged. In the digestive tracts of these flies, RFP and GFP signals were observed in the crop, midgut, and hindgut regions (Figure 2b). This observation implies effective ingestion of GMNL-185 and GMNL-680 by flies. Next, we investigated whether GMNL-185 and GMNL-680 survived in the fly digestive tract. After 5 days of GMNL-185 or GMNL-680 feeding, digestive tracts were isolated from flies and then homogenized in phosphate-buffered saline (PSB). The supernatant was spread onto MRS agar plates and incubated at 37°C for 2–3 days. The colonies that emerged on the plate exhibited robust expression of RFP or GFP (Figure 2c). Collectively, these findings indicate the presence of viable GMNL-185 and GMNL-680 within the digestive tract of flies.

(a) Confocal imaging of the fly digestive tract. The samples were immunostained with the anti-βPS-integrin antibody (gray). Scale bar = 500 μm. The fly digestive tract can be subdivided into distinct regions, including the foregut, midgut, and hindgut. Midgut can be further subdivided into R1–R5 regions. (b) RFP-tagged GMNL-185 or GFP-tagged GMNL-680 strains were cultured in *Lactobacillus*-supplemented fly food medium. Wild-type flies were fed with food medium containing RFP-tagged GMNL-185 or GFP-tagged GMNL-680 for five days, and their digestive tracts were dissected and mounted on coverslips. RFP and GFP fluorescent signals were detected in the crop, R1, R2, R3, R4, R5, and hindgut sub-regions of their digestive tracts. In contrast, the control group flies were fed with regular food without *Lactobacillus* spp. for five days. No RFP and GFP signals were observed in the digestive tracts of this group. Scale bar = 50 μm. (c) After five days of feeding with a medium containing RFP-tagged GMNL-185 or GFP-tagged GMNL-680, the fly digestive tracts were isolated and homogenized in PBS. The resulting supernatants were spread on MRS agar plates and incubated at 37°C for 2–3 days. Colonies of RFP-tagged GMNL-185 (c_1_) and GFP-tagged GMNL-680 (c_3_) were cultured on MRS agar plates. Individual colonies were isolated from the cultured MRS agar plates and placed on coverslips for confocal microscopy. The cultured bacterial colonies exhibited distinct RFP fluorescence (c_2_) and GFP fluorescence (c_4_) under confocal microscopy. Scale bar = 20 μm.

### Synergistic effects of mixed GMNL-185 and GMNL-680 feeding on memory functions in flies

**Figure 3.**
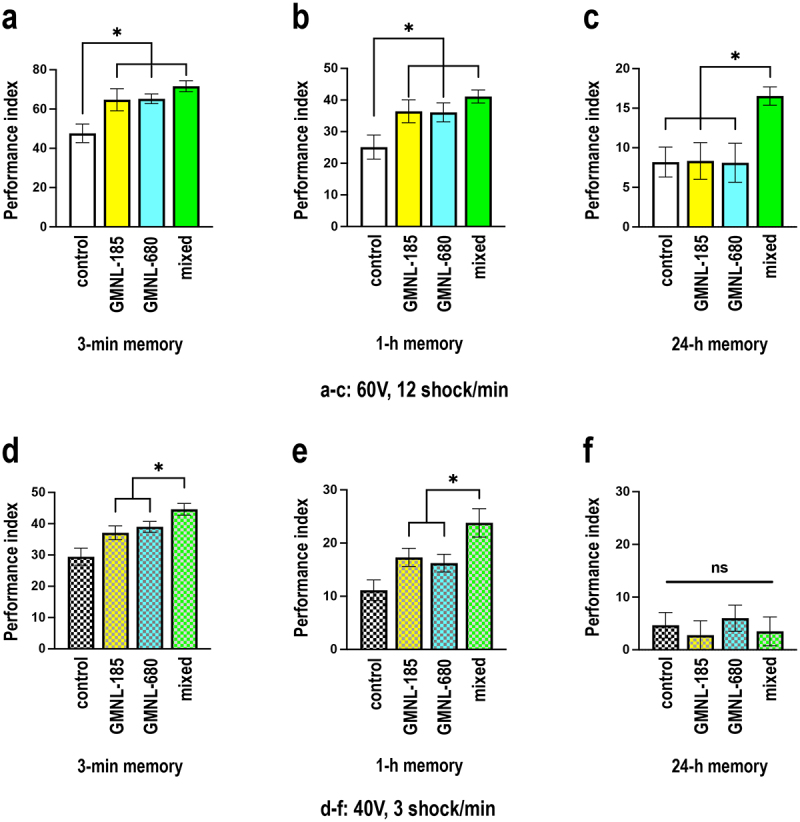
Flies with GMNL-185 and GMNL-680 mixed feeding showed synergistic effects on olfactory memory.

The lyophilized live probiotic powder, containing GMNL-185 and GMNL-680 *Lactobacilli* (3 × 10^9^ CFU/mL), were added to the fly food. Flies were orally administered the probiotic powder with fly food for 5 days. An equal amount of corn powder was added to fly food as the control group. We investigated the memory performance in these flies and the results showed no significant differences in the 3-minute memory and 1-hour memory between the mixed strain-fed flies and those fed with a single strain (Figure 3(a,b)). In terms of 24-hour memory, flies in the mixed feeding group exhibited a noteworthy enhancement compared to the control group or the single-strain probiotic group (Figure 3c). Previous studies have shown that olfactory memory performance exhibits a ceiling effect when associated with a 1-minute odor and 12-time 60-volt electric shocks.^19,50,51^ We further investigated whether the lack of significant differences in 3-minute and 1-hour memory enhancement between the mixed feeding and single strain feeding groups was due to the ceiling effect. We employed a milder training protocol for subsequent experiments in which flies were trained to associate a 1-minute odor with only three occurrences of 40-volt electric shocks. Results showed significantly enhanced 3-minute and 1-hour memories in the mixed feeding group compared to the control or the single-strain probiotic feeding groups (Figure 3(d,e)). However, no significant differences in 24-hour memory were observed between the different groups (Figure 3f). Taken together, these findings suggest that mixed feeding with the two different *Lactobacillus* stains has a synergistic effect on memory performance in flies. Since live GMNL-185 and GMNL-680 strains were found in the fly digestive tract five days after probiotics feeding (Figure 2), we then asked whether memory improvement effects observed in our fly groups are due to live microorganisms. To address this issue, we autoclaved the GMNL-185 and GMNL-680 mixed powder at 121°C for 30 minutes before administering it to flies. Interestingly, this group of flies did not exhibit enhanced 3-minute memory after five days of feeding (Figure S1). These results suggest that the beneficial effects of GMNL-185 and GMNL-680 are due to the presence of live stains in the fly digestive tract (Figure S1).

(a) In the regular training protocol (12 instances of 60-volt electric shock with CS+ odor for 1 minute), flies showed enhanced 3-minute memory performance in the GMNL-185 or GMNL-680 feeding group. The mixed feeding group (GMNL-185 + GMNL-680) did not exhibit significant differences from the GMNL-185 or GMNL-680 feeding groups. Each bar represents the mean ± SEM (*N* = 8), *p < 0.05; one-way ANOVA followed by Tukey’s test. (b) Flies showed enhanced 1-hour memory performance in GMNL-185 or GMNL-680 feeding groups. The mixed feeding group showed no significant difference from the GMNL-185 or GMNL-680 feeding groups. Each value represents the mean ± SEM (*N* = 9), *p < 0.05; one-way ANOVA followed by Tukey’s test. (c) Flies did not show enhanced 24-hour memory performance in GMNL-185 or GMNL-680 feeding groups as compared to the control group; however, enhanced 24-hour memory performance was observed in the mixed feeding group. Each bar represents the mean ± SEM (*N* = 9‒15), *p < 0.05; one-way ANOVA followed by Tukey’s test. (d) In the milder training protocol (three times 40-volt  electric shock plus CS+ odor for 1 minute), flies showed enhanced 3-minute memory performance in GMNL-185 or GMNL-680 feeding groups, while the mixed feeding group showed a synergistic effect. Each value represents the mean ± SEM (*N* = 13‒18), *p < 0.05; one-way ANOVA followed by Tukey’s test. (e) In milder training conditions, flies showed enhanced 1-hour memory performance in GMNL-185 or GMNL-680 feeding groups as compared to the control group. The mixed feeding group exhibited a synergistic effect. Each value represents the mean ± SEM (*N* = 15‒22), *p < 0.05; one-way ANOVA followed by Tukey’s test. (f) In milder training conditions, flies did not show a significant enhancement of 24-hour memory performance in GMNL-185, GMNL-680, or mixed feeding groups as compared to the control group. Each value represents the mean ± SEM (*N* = 8‒9). Non-significant difference (ns), *p* > 0.05; one-way ANOVA.

### Increased cellular calcium responses to training odor in mushroom body β and γ lobes of GMNL-185 and GMNL-680 mixed feeding group flies

**Figure 4.**
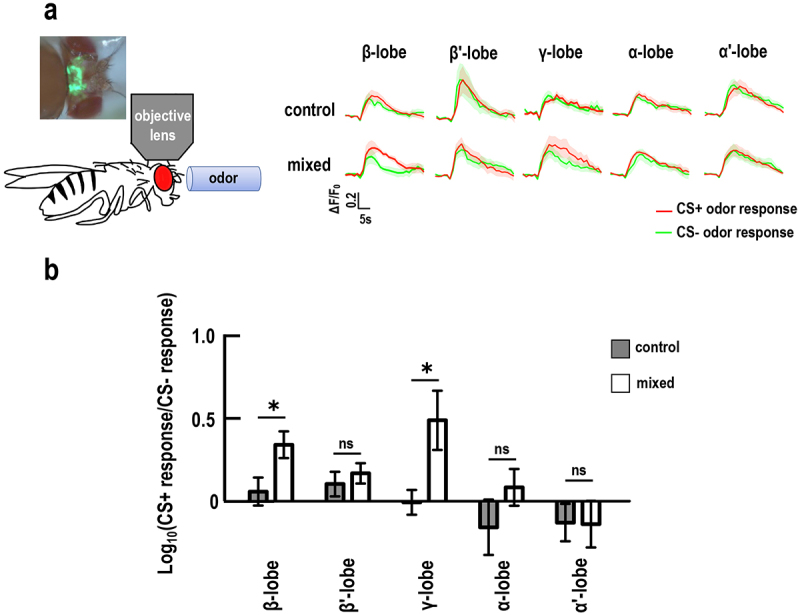
Flies with GMNL-185 and GMNL-680 mixed feeding showed increased calcium responses to training odor in the mushroom body β and γ lobes.

It has been reported that flies form learning-induced plasticity changes (called memory traces) after association with a particular CS+ odor (training odor) with electric shock, which alters neuronal activity in response to the CS+ odor.^52,53^ Ample studies have shown that memory traces occur in the mushroom body, which can be visualized by functional optical *in vivo* calcium imaging in living flies.^54–57^ Therefore, we investigated the neuronal activity in the mushroom body in response to the CS+ odor after training via the functional calcium imaging, in flies subjected to GMNL-185 and GMNL-680 mixed feeding. Results showed that mixed feeding group flies exhibited the most significant memory enhancement in 1-hour after the milder training protocol. Next, we investigated calcium responses to training odor 1-hour after milder training. Flies were fed with mixed GMNL-185 and GMNL-680 *Lactobacillus* spp. for five days and then trained to associate an odor with three-time 40-volt electric shocks (milder learning protocol) after which live calcium brain imaging was performed at 1-hour after training. Flies carrying *UAS-GCaMP6m* and *R13F02-GAL4* (GAL4-labeled mushroom body neurons) were fed mixed GMNL-185 and GMNL-680 microbes and then assigned to different experimental groups. In the control group, *UAS-GCaMP6m*; *R13F02-GAL4* flies were fed an equivalent amount of cornmeal powder. The mushroom body comprises of γ, αβ, and α′β′ neurons, and the recordings were performed on optical sections in regions of the vertical (α′ and α) and horizontal (β′, β, and γ) lobes as illustrated (Figure S2). Results showed a significant increase in calcium responses to training odor within γ and β lobes of the mushroom body 1 hour after the milder training (Figure 4).

(a) The GCaMP6 responses to the training odor were assessed in distinct mushroom body lobes of GMNL-185 and GMNL-680 mixed feeding group flies. The calcium responses of GCaMP6 were measured 1 hour following a milder training protocol (consisting of 40 volts and 3 episodes of electric shocks within 1 minute) in each mushroom body lobe. The GMNL-185 and GMNL-680 mixed feeding group flies exhibited enhanced calcium responses to the training odor (CS+) compared to the non-training odor (CS−), with a notable increase observed in the β and γ lobes. This increase in calcium responses to the CS+ odor was not observed in the control group (without mixed GMNL-185/GMNL-680 feeding). (b) Quantification of the GCaMP6 responses to the CS+ odor relative to the CS− odor at 1 hour following a milder training protocol. The different regions of the mushroom body lobes were analyzed, respectively. The logarithmic ratios of the CS+ response to the CS− response were computed using the peak response amplitudes. Each value represents the mean ± SEM (*N* = 12‒20). *p < 0.05; non-significant differences (ns), *p* > 0.05, analyzed using Student’s *t*-test.

To further validate the physiologic effects of the probiotic feeding flies are indeed caused by GMNL-185 and GMNL-680 administration, we therefore used *L. rhamnosus* #2 as the negative bacterial control in our study. We found that *L. rhamnosus* #2 were survived in fly’s digestive tracts after 5 days of probiotic-containing food feeding (Figure S3). However, the existence of *L. rhamnosus* #2 in fly’s digestive tracts had no effects on 3-minute memory (Figure 1), 1-hour memory (Figure S4a and S4b), or the neuronal responses to CS+ odor in the mushroom body after training (Figure S4c-S4e).

### GMNL-185 and GMNL-680 mixed feeding increases lactate dehydrogenase, pyruvate dehydrogenase, and choline acetyltransferase levels in the mushroom body

**Figure 5.**
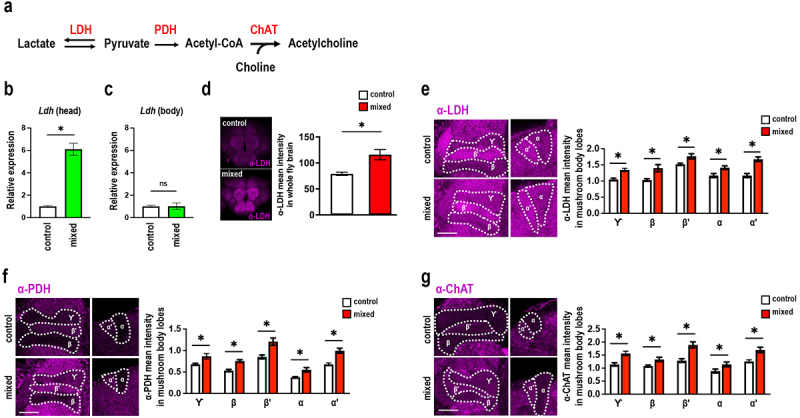
GMNL-185 and GMNL-680 mixed feeding flies showed an increased LDH level in the brain but not in the body.

Lactic acid is produced via fermentation of carbohydrates by different microorganisms, such as the *Lactobacillus* spp. The enzyme LDH facilitates the interconversion of lactate and pyruvate (Figure 5a). Previous studies have highlighted the significance of neuronal and glial *Ldh* expression in maintaining long-term courtship memory during aging.^37,58^ Therefore, we examined the expression levels of *Ldh* in flies subjected to mixed feeding of GMNL-185 and GMNL-680 by quantitative reverse transcription polymerase chain reaction (qRT-PCR). Results showed that *Ldh* mRNA levels were significantly increased in the head of adult flies in the GMNL-185 and GMNL-680 mixed feeding group compared to those in the control group (Figure 5b). Noticeably, there were no significant changes in *Ldh* mRNA levels in the bodies of flies in different groups (Figure 5c).

(a) Scheme of enzymes involved in converting lactate into acetylcholine (b) *Ldh* mRNA levels were significantly increased in the heads of GMNL-185 and GMNL-680 mixed feeding group flies (Student *t*-test, *p < 0.05). Each value represents the mean ± SEM (*N* = 4). (c) *Ldh* mRNA levels were not significantly changed in the body of GMNL-185 and GMNL-680 mixed feeding group flies. Each value represents the mean ± SEM (*N* = 6‒8). (d) Quantification of LDH protein level in the whole fly brain after GMNL-185 and GMNL-680 mixed feeding. The brain structures were immunostained with anti-LDH antibody (magenta). Flies showed increased brain LDH expression in the GMNL-185 and GMNL-680 mixed feeding group (Student’s *t*-test, *p < 0.05). Each value represents the mean ± SEM (*N* = 8‒9). The fly brains were immunostained with (e) anti-LDH antibody, (f) anti-PDH antibody, and (g) anti-ChAT antibody (magenta). There were significant increased LDH, PDH, and ChAT immunostaining signals in mushroom body lobes in GMNL-185 and GMNL-680 mixed feeding flies as compared to the control flies. The intensity of immunostaining signals in mushroom body lobes from anti-LDH, anti-PDH, or anti-ChAT antibodies were normalized to the intensity of immunostaining signals in subesophageal ganglion (SOG) from the same antibody. Each value represents the mean ± SEM (*N* = 8‒10); Student’s *t*-test, *p < 0.05.

To further validate brain LDH protein levels in GMNL-185 and GMNL-680 mixed feeding group flies, we conducted whole-mount brain immunohistochemistry using anti-LDH antibody. Results showed a significant increase in brain LDH protein levels in flies of the mixed feeding group (Figure 5d). Since the mushroom body plays a critical role in olfactory memory formation,^20–26^ we further investigated LDH protein expression in different mushroom body lobes (Figure 5e, left panel). Results confirmed increased LDH protein levels within the whole mushroom body in flies who received GMNL-185 and GMNL-680 mixed feeding, compared to the control group flies (Figure 5e, right panel). Moreover, pyruvate is a key precursor of acetyl-CoA, which is oxidized for energy production in the TCA cycle.^59^ Hence, we investigated PDH expression levels, the key enzyme for converting pyruvate to acetyl-CoA,^59^ in the mushroom body lobes. Our results showed significant increases of PDH protein levels in mushroom body lobes in GMNL-185 and GMNL-680 mixed feeding flies as compared to the control flies (Figure 5f). Additionally, it has been shown that acetylcholine is the major neurotransmitter in mushroom body for olfactory memory in *Drosophila*.^60^ Acetylcholine is synthesized in neurons from two precursors, the acetyl-CoA and choline, in a reaction catalyzed by an enzyme called choline acetyltransferase (ChAT).^61^ We therefore quantified the ChAT protein expression levels in the mushroom body after GMNL-185 and GMNL-680 mixed feeding. Our results showed a significant increase in ChAT protein levels in mushroom body lobes after GMNL-185 and GMNL-680 mixed feeding as compared to the control group (Figure 5g).

### Silencing *Ldh* in the mushroom body disrupts the memory improvement effects in GMNL-185 and GMNL-680 mixed feeding flies

**Figure 6.**
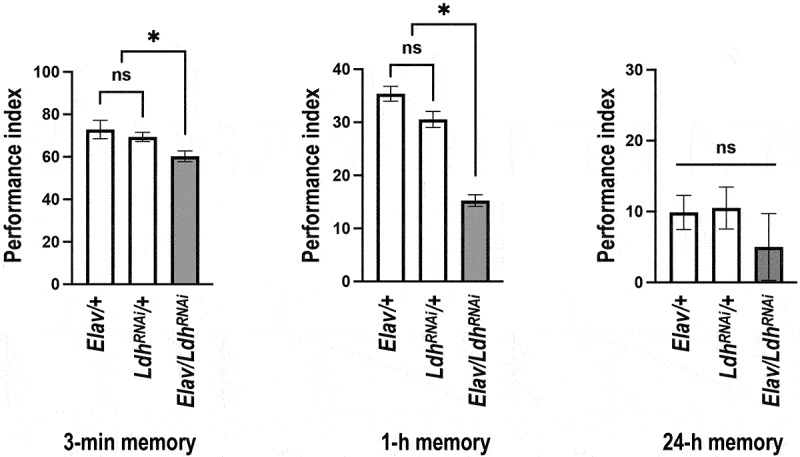
Pan-neuronal knockdown of *Ldh* impairs GMNL-185/GMNL-680 mixed-feeding-induced memory enhancement.

**Figure 7.**
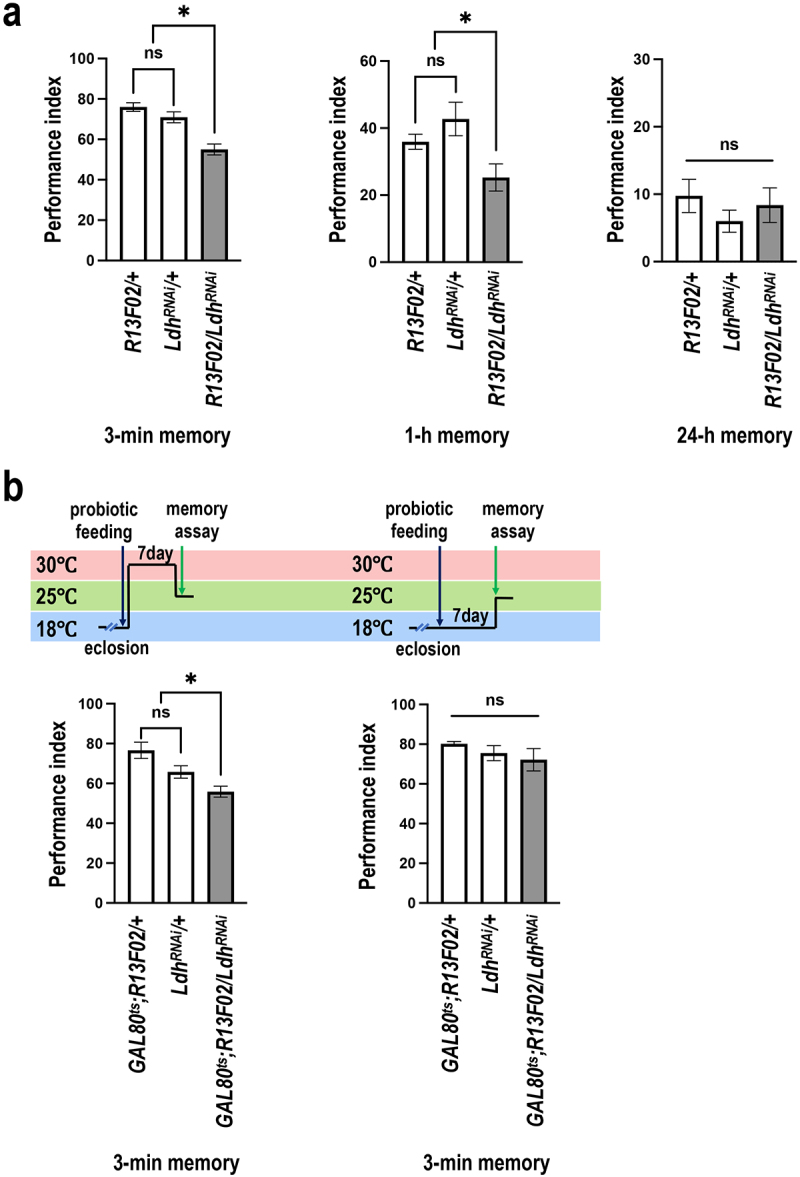
*Ldh* knockdown specifically in the mushroom body impairs GMNL-185/GMNL-680 mixed-feeding-induced memory enhancement.

**Figure 8.**
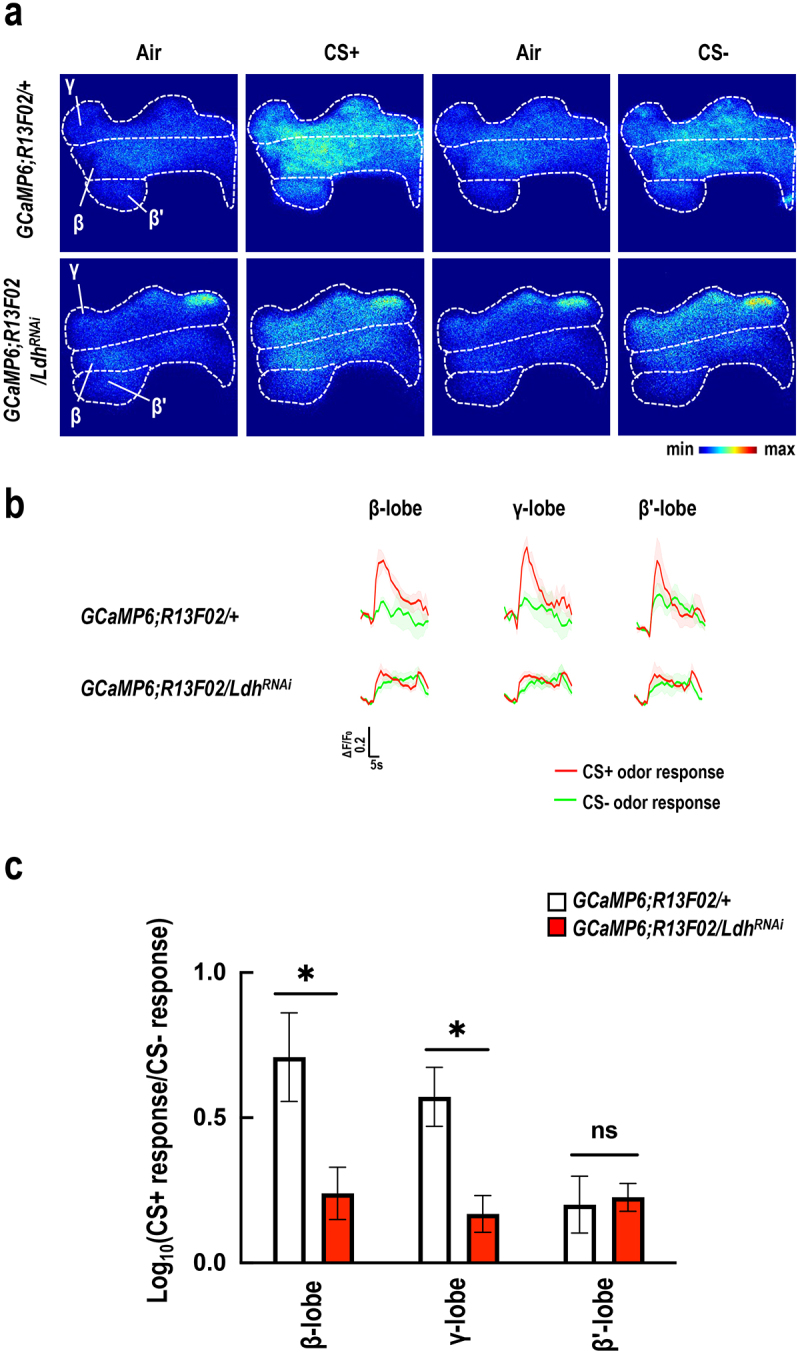
*Ldh* knockdown abolished the GMNL-185/GMNL-680 mixed-feeding-induced increased responses to training odor in the mushroom body β and γ lobes.

**Figure 9.**
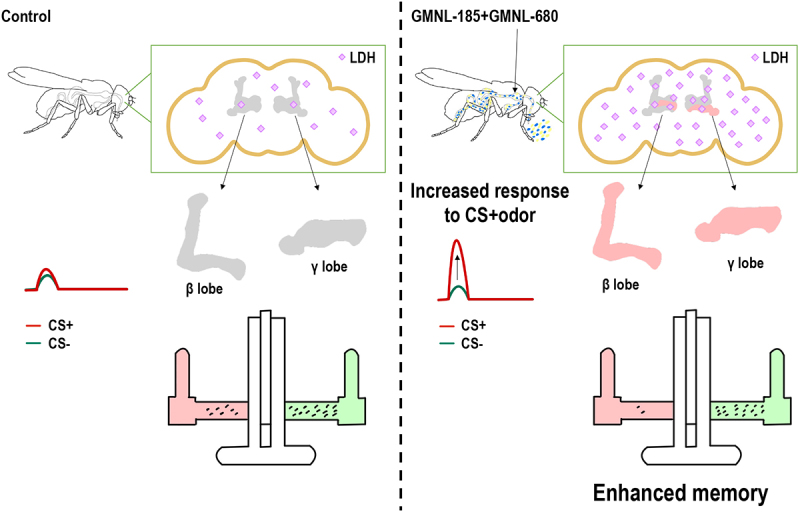
Proposed model showing the effect of GMNL-185/GMNL-680 mixed feeding on *Drosophila* memory.

Our findings suggest that neuronal LDH might play a pivotal role in olfactory memory (Figure 5). To confirm this, we performed pan-neuronal *Ldh* knockdown by expressing *Ldh*^*RNAi*^ using *Elav- GAL4*, a neuronal driver of GAL4. Behavioral assays were conducted following a 5-day regimen of GMNL-185 and GMNL-680 mixed feeding. Results showed that pan-neuronal *Ldh* knockdown abrogated the memory-enhancing effects of GMNL-185 and GMNL-680 (Figure 6). Since LDH levels were also increased in mushroom body neurons, we further knocked down *Ldh* expression in mushroom body neurons via *R13F02-GAL4* driven *UAS-Ldh*^*RNAi*^. Results showed that targeted knockdown of *Ldh* expression in the mushroom body has similar effects (Figure 7a). To exclude the potential developmental effects caused by the chronic expression of *Ldh*^*RNAi*^, we introduced *tub-GAL80*^*ts*^ for temporal control of *Ldh*^*RNAi*^ expression in mushroom body specific to the adult stage. Flies were incubated at 18°C during development and were shifted to 30°C for seven days after eclosion. Behavioral assays were performed at 25°C. We found that the 3-minute memory was disrupted in probiotic-fed flies carrying *R13F02-GAL4; tub-GAL80*^*ts*^ > *UAS-Ldh*^*RNAi*^ transgenes (Figure 7b, left panel). However, this disruption was not observed in flies incubated at 18°C throughout development (Figure 7b, right panel). Live brain imaging data showed that genetic knockdown of *Ldh* expression abolished the GMNL-185/GMNL-680 mixed-feeding-induced calcium responses to CS+ odor in the mushroom body β and γ lobes (Figure 8). These results confirmed that GMNL-185/GMNL-680 mixed feeding upregulates LDH expression in the mushroom body β and γ lobes, thereby regulating memory traces in flies (Figures 3–5, 7 –9).

All flies were fed a mixture of GMNL-185 and GMNL-680 for five days. *Elav-GAL4 > UAS-Ldh*^*RNAi*^ flies exhibited impaired performance in 3-minute memory and 1-hour memory compared to *Elav-GAL4 > +* and *UAS-Ldh*^*RNAi*^ *> +* flies. *Elav-GAL4 > UAS-Ldh*^*RNAi*^ flies. *Elav-GAL4 > UAS-Ldh^R^*^*NAi*^ flies did not exhibit impaired performance in 24-hour memory compared to *Elav-GAL4 > +* and *UAS-Ldh*^*RNAi*^ *> +* flies. Each value represents the mean ± SEM (*N* = 8). *p < 0.05; non-significant differences (ns), *p* > 0.05, analyzed using one-way ANOVA followed by Tukey’s test.

All flies were fed with a mixture of GMNL-185 and GMNL-680 for five days before the behavioral assays. (a) *R13F02-GAL4 > UAS-Ldh*^*RNAi*^ flies exhibited disrupted performance in the 3-minute memory (*N* = 13) and 1-hour memory (*N* = 11) tests compared to *R13F02-GAL4 > +* and *UAS-Ldh*^*RNAi*^ *> +* flies. Moreover, 24-hour memory was not affected in *R13F02-GAL4 > UAS-Ldh*^*RNAi*^ flies compared to that in *R13F02-GAL4 > +* and *UAS-Ldh*^*RNAi*^ *> +* flies (*N* = 8). Each value represents the mean ± SEM. *p < 0.05; one-way ANOVA followed by Tukey’s test. (b) Adult stage-specific knockdown of *Ldh* in the mushroom body disrupts memory. Acute knockdown of *Ldh* expression in the entire mushroom body neurons using *R13F02-GAL4* impaired the 3-minute memory. Adult flies were reared at 18°C and then shifted to 30°C for seven days before training to recover from *tub-GAL80*^*ts*^ inhibition of the *GAL4* activity. Each value represents the mean ± SEM (*N* = 6), *p < 0.05; non-significant difference (ns), *p* > 0.05, determined using one-way ANOVA followed by Tukey’s test.

(a) All flies were fed with GMNL-185/GMNL-680 for five days before behavioral assays. The calcium responses of GCaMP6 were measured 1 hour following a milder training protocol in each mushroom body lobe. Recordings were made from optical sections in regions of the horizontal (β’, β, and γ lobes) of the mushroom body. (b) Flies bearing *UAS-GCaMP6m/+; R13F02-GAL4/+* showed significantly increased calcium responses to CS+ odor in the mushroom body β and γ lobes after milder training protocol, but no changes were observed in flies carrying *UAS-GCaMP6m/+*; *R13F02-GAL4/UAS-Ldh*^*RNAi*^. (c) Quantification of the GCaMP6 responses to the CS+ odor relative to the CS− odor at 1 hour following a milder training protocol. The different regions of the mushroom body lobes were analyzed, respectively. The logarithmic ratios of the CS+ response to the CS− response were computed using the peak response amplitudes. Each value represents the mean ± SEM (*N* = 7‒9). *p < 0.05; non-significant difference (ns), *p* > 0.05, determined using Student’s *t*-test.

Flies that were fed with a mixture of GMNL-185 and GMNL-680 exhibited elevated *Ldh* levels within the brain and mushroom body neurons, along with increased calcium responses in the mushroom body β and γ lobes. Consequently, flies exhibited enhanced olfactory memory after GMNL-185 and GMNL-680 feeding, which was abolished by silencing *Ldh* expression in the mushroom body. Our results demonstrate potent probiotic effects of the two *Lactobacillus* spp. which improves the memory performance in flies through the gut-brain axis and remarkably enhances neuronal LDH levels in the brain.

Next, we investigated whether pan-neuronal or mushroom body-specific overexpression of *Ldh* could enhance memory performance in flies. To address this, memory performance was assessed in flies carrying *Elav-GAL4 > UAS-Ldh* or *R13F02-GAL4 > UAS-Ldh*. Results showed that pan-neuronal or mushroom body-specific *Ldh* overexpression does not affect memory performance in flies (Figures S5 and S6).

## Discussion

Previous studies have indicated a correlation between the gut microbiota, emotional behaviors, and cognitive functions of the host.^12,62^ In *Drosophila*, studies have highlighted the significant impact of the gut microbiota on memory and sleep homeostasis.^63,64^ Moreover, studies on the effects of oral probiotics on brain functions revealed that flies possess a well-integrated gut-brain axis,^65^ rendering them a potential model for studying gut microbiota interactions.^15,18^ Our study was designed to explore the effect of *Lactobacillus* spp. feeding on memory enhancement in *D. melanogaster*. We found that in files fed with *Lactobacillus* spp. GMNL-185 or GMNL-680, the olfactory memory is enhanced, suggesting beneficial effects of these gut microbes. Next, we investigated whether co-feeding with both strains has a synergistic effect. Previous studies suggest that multi-strain mixtures do not exhibit significant superiority over single-strain approaches in most cases.^66^ However, some studies showed that multi-strain probiotics enhanced benefits due to the strains’ synergy or additively.^66,67^ We demonstrated synergistic effects of GMNL-185 and GMNL-680 co-feeding on memory functions in flies. Moreover, the absence of memory enhancement effects in flies fed with dead GMNL-185 and GMNL-680 suggests that the beneficial effects of the gut *Lactobacillus* spp. are dependent on live bacteria. The enhanced olfactory responses to the training odor observed in the mushroom body γ and β lobes of mixed GMNL-185 and GMNL-680 fed flies indicate that the enhanced olfactory memory is due to the increased mushroom body neuronal activity (Figure 3). GMNL-185 and GMNL-680 are known to improve bacterial vaginosis by which GMNL-185 inhibits the growth of *Gardnerella vaginalis* (*G. vaginalis*) and GMNL-680 inhibits *Streptococcus agalactiae* (*S. agalactiae*) and *Staphylococcus aureus* (*S. aureus*).^68^ Moreover, GMNL-185 plays roles for modifying the gut microbiota to decrease the levels of depression-related metabolites^69^ or inhibit the growth of *Helicobacter pylori* (*H. pylori*) in mice, thereby, ameliorating inflammation in stomach.^70^ GMNL-185 strain has potentials of anti-depressive and treated of gastrointestinal diseases. Together with previous findings, our results suggest that GMNL-185 and GMNL-680 could act synergistically not only to prevent bacterial infections in mice but also to enhance memory functions in fruit flies.

It has been shown that aversive short-term memory is formed in the mushroom body γ neurons, whereas long-term memory is formed in mushroom body αβ neurons via cAMP signaling.^71^ Our results suggest that increased calcium responses in the mushroom body γ and β lobes could represent short-term and long-term memory traces, respectively. We further showed that LDH protein levels are increased specifically in brain neurons after GMNL-185 and GMNL-680 mixed feeding. LDH regulates the reversible conversion of lactate to pyruvate, a critical step in metabolic pathways. GMNL-185 and GMNL-680 belong to the Lactobacillaceae family and are known to produce lactic acid as a major product. However, based on our results, we were unable to conclude whether lactic acid production in the fly digestive tract upregulates *Ldh* levels in the brain. To investigate the role of brain *Ldh* expression on memory enhancement, we performed pan-neuronal silencing of *Ldh* using *Elav-GAL4* > *UAS-Ldh^RNAi^* and then conducted behavioral assays on flies. Results showed that silencing *Ldh* in neurons abolished the memory-enhancing effects of GMNL-185 and GMNL-680 mixed feeding. These results indicate that co-feeding GMNL-185 and GMNL-680 significantly increases LDH levels in the fly brain, which in turn enhances olfactory memory.

Lactate is converted to pyruvate by LDH and can be further converted to acetyl-CoA by the PDH under aerobic conditions. Acetyl-CoA is the source of acetylcholine, a major neurotransmitter in mushroom body neurons.^60^ Blocking acetylcholine biosynthesis in the mushroom body causes olfactory memory defects in *Drosophila*.^60^ Our findings revealed increased LDH levels in the mushroom body following GMNL-185 and GMNL-680 mixed feeding, suggesting that enhanced LDH levels could potentially promote acetyl-CoA generation resulting in elevated acetylcholine levels within mushroom body neurons which is critical for memory formation.^60^ The increased ChAT protein levels in mushroom body after GMNL-185 and GMNL-680 mixed feeding further support this notion that elevated acetylcholine levels in the mushroom body after probiotic administration (Figure 5). Altogether, it suggests that GMNL-185 and GMNL-680 mixed feeding increases LDH, PDH, and ChAT protein expression levels, finally strengthening the acetylcholine neurotransmission in mushroom body, thereby enhancing olfactory memory. The abrogation of the beneficial effects of GMNL-185 and GMNL-680 mixed feeding on olfactory memory upon *Ldh* silencing within the mushroom body further supports this conclusion (Figure 7). Additionally, GMNL-185 and GMNL-680-fed flies exhibited significantly increased calcium responses to CS+ odor in the mushroom body γ and β lobes after the milder training protocol (Figure 4). These findings suggest that probiotic feeding enhances the responses to the CS+ odor during memory retrieval, thereby contributing to enhanced olfactory memory.

Since we observed elevated LDH protein levels in both the brain and the mushroom body neurons following 5 days of GMNL-185 and GMNL-680 mixed feeding, we evaluated the effect of *Ldh* knockdown on memory. Notably, silencing *Ldh* in neurons or the mushroom body not only abolished the memory enhancement effects of mixed feeding but also reduced the responses to CS+ odor after the training (Figures 7 and 8). Together, these results imply that GMNL-185 and GMNL-680 mixed feeding increases LDH expression, thereby contributing to memory and memory traces improvement (Figures 7–9). However, *Ldh* overexpression in neurons or the mushroom body did not affect memory functions (Figures S5 and S6). Furthermore, no memory-enhancing effects of dead GMNL-185 and GMNL-680, suggesting that live *Lactobacillus* spp. are required for memory enhancement (Figure S1). Our results also indicate the potential involvement of unknown metabolites from GMNL-185 and GMNL-680 in memory improvement in flies. However, this speculation needs further investigation in future studies.

## Materials and methods

### Fly stocks

Flies were reared on standard cornmeal food and kept at a constant temperature of 25°C and 60% relative humidity under a 12-hour light/12-hour dark cycle. Wild-type flies used in this study were of the *w^1118^* (*isoCJ1*) Canton-S strain. For modulating gene expression, the following GAL4 and UAS strains were obtained from the Bloomington Drosophila Stock Center (BDSC): *R13F02-GAL4* (BDSC#48571), *Elav-GAL4* (BDSC#8765), *tub-GAL80*^*ts*^ (BDSC#7019), *UAS-Ldh* (BDSC#16829), *UAS-GCaMP6m* (BDSC#42748), and *UAS-Ldh*^*RNAi*^ (BDSC#33640).

### Bacterial culture and *Lactobacillus* spp. feeding

Lactobacilli used in this study were provided by GenMont Biotech (Tainan, Taiwan), including *L. plantarum* (#1–4), *L. casei* (#1), *L. paracasei* (#1–3), *L. rhamnosus* (#1–3), *L. reuteri* (#1), *L. fermentum* (#1–2), *L. acidophilus* (#1). *Lactobacillus* spp. were cultured in MRS (BD, Franklin Lank, NJ, USA) broth and agar plates at 37°C overnight. The OD600 of the bacterial culture was then adjusted to 1.0 (approximately 5 × 10^8^ cells/mL). One hundred microliters of the bacterial culture were added to the fly medium (25% MRS culture medium and 75% standard fly food without antibiotics). This medium was used during the initial 3-minute memory screening.

Corn powder containing a single *Lactobacillus* strain, the GMNL-185 (BCRC 910,774; CCTCC M 2,017,764), the GMNL-680 (BCRC 910,775; CCTCC M 2,017,766), or the *L. rhamnosus* #2 was obtained from GenMont Biotech Inc. The *Lactobacillus*-supplemented food mixture consisted of 25% MRS culture medium and 75% standard fly food (without antibiotics) along with *Lactobacillus* powder (3 × 10^9^ CFU/mL) or an equivalent amount of corn powder (control). Flies were collected three days after eclosion and transferred to the *Lactobacillus*-supplemented food. The flies were maintained at 30°C and 60% relative humidity for the next five days.

### Transformation

*Lactobacillus* spp. (GMNL-185, GMNL-680, and *L. rhamnosus* #2) were cultured overnight in MRS broth supplemented with 5% threonine, after which the culture was transferred into a pre-warmed MRS broth supplemented with 5% threonine until the OD600 reached 0.6–0.8. Subsequently, the cells were centrifuged at 8000 rpm and 4°C, followed by three rounds of washing with cold electroporation buffer (0.286 M sucrose, 20% glycerol, and 1 mM MgCl_2_). The cells were then resuspended in 1 mL of cold electroporation buffer. A mixture comprising 100 µL of the cell suspension and 2 µg of the plasmid DNA (*pTRKH3-ermGFP* or *pLEM415-ldhL-mRFP1*) was placed into 2 mm gap electroporation cuvettes (BioRad). The electroporation conditions were as follows: 2.5 kV, 200 Ω, and 25 µFD. Following electroporation, the cells were incubated in MRS broth for 3 h at 37°C. The cells were then plated on MRS agar plates containing 10 µg/mL erythromycin and incubated under anaerobic conditions at 37°C for 2–3 days. The OD600 of bacterial culture was adjusted to 1 (approximately 5 × 10^8^ cells/mL), and 100 μL of this culture was added to the fly medium.

### Analysis of the gut microbiota

To investigate whether *Lactobacillus* spp. (GMNL-185, GMNL-680, and *L. rhamnosus* #2) could colonize the digestive tract of *Drosophila*, we dissected out the entire digestive tract from 10–15 female flies in sterile PBS. The dissected tissues were thoroughly homogenized in 100 μL sterile PBS using small pestles. Subsequently, 50 μL of sterile PBS was added and the solution was centrifuged for 5 min at 1000 rpm and 4°C. Then, 100 μL of the resulting supernatant (either 1/10 diluted or undiluted) was spread onto agar plates and incubated at 37°C for 2–3 days. Once colonies became visible on the agar plate, a long coverslip was used to spread the colonies gently, and then a smaller coverslip was placed over the colony for imaging using the Zeiss LSM 700 confocal microscope equipped with a 40× C-Apochromat water-immersion objective (N.A. = 1.2, working distance = 220 μm).

### Staining and imaging

Samples (brain or digestive tract) were isolated in isotonic PBS and immediately transferred to 4% paraformaldehyde (PFA) for 20 min fixation. The samples were then incubated in PBS containing 2% Triton X-100 and 10% normal goat serum for 2 h at 25°C. After 2 h of penetration and blocking, the samples were degassed. After, the digestive tracts were incubated in 1:100 diluted anti-βPS-integrin mouse monoclonal antibody (CF.6G11; Developmental Studies Hybridoma Bank, University of Iowa) and the brains were incubated in 1:200 diluted polyclonal rabbit anti-LDH (PA5–26531; Thermo Fisher Scientific) or 1:500 diluted monoclonal mouse anti-PDH (ab110334; abcam) or 1:200 diluted monoclonal mouse anti-ChAT (ChAT4B1; Developmental Studies Hybridoma Bank, University of Iowa) at 25°C for 24 h. After washing 3 times in PBS containing 1% Triton X-100 (PBS-T), the digestive tracts were incubated in biotinylated goat anti-mouse IgG (1:200; B-2763, Thermo Fisher Scientific) and the brains were incubated in biotinylated goat anti-rabbit IgG (1:200; B-2770, Thermo Fisher Scientific) or biotinylated goat anti-mouse IgG (1:200; B-2763, Thermo Fisher Scientific) at 25°C for 24 h. The samples were then washed and incubated in Alexa Fluor 633 streptavidin or Alexa Fluor 647 streptavidin (1:500; Invitrogen) at 25°C for 24 h. The samples were washed three times with PBS-T, cleared, and mounted in FocusClear (CelExplorer) for imaging. Images were obtained using the Zeiss LSM 700 confocal microscope.

### Olfactory memory assays

Groups of approximately 100 flies were initially exposed to the first odor (CS+) which was paired with either 12 instances of a 1.5 s pulse of 60 volts DC electric shock or 3 instances of a 1.5 s pulse of 40 volts DC electric shock over 1 min. Subsequently, the flies were exposed to a second odor (CS–) without any electric shock for an additional 1 min. The specific odors used in these experiments were 3-octanol (OCT; Sigma-Aldrich, product no. 218405) and 4-methylcyclohexanol (MCH; Sigma-Aldrich, product no. 153095). For the testing phase, flies were given 2 min to choose between the CS+ and CS– odor tubes within a T-maze setup. To assess the duration of the memory, trained flies were tested at 3 min, 1 h, and 24 h post-training. For calculating the performance index, the number of flies in the CS– odor chamber was subtracted from the number of flies in the CS+ odor chamber, and the resulting value was divided by the total number of flies. For adult stage-specific knockdown of *Ldh*, the *tub-GAL80*^*ts*^ system was employed. Flies were reared at 18°C until eclosion, after which they were transferred to *Lactobacillus*-supplemented food and maintained at 30°C for 7 days prior to the olfactory conditioning. The control group of flies was kept at 18°C throughout the entire experimental duration.

### Live calcium brain imaging

Flies carrying *UAS-GCaMP6m; R13F02-GAL4* transgenes were reared on *Lactobacillus*-supplemented food at 30°C for five days prior to odor/electric shock training. The flies underwent milder training, which involved three instances of a 40-volt electric shock paired with an odor. Calcium imaging was performed 1 h after each training session. To prevent dehydration, the flies were gently held within a 250 mL pipette tip, a small hole was made in the head, and a drop of adult hemolymph-like (AHL) saline (108 mM NaCl, 5 mM KCl, 2 mM CaCl_2_, 8.2 mM MgCl_2_, 4 mM NaHCO_3_, 1 mM NaH_2_PO_4_, 5 mM trehalose, 10 mM sucrose, and 5 mM HEPES; pH 7.5, 265 mOsm) was introduced. After clearing the cerebral trachea and fat, the pipette tip (including the fly) was securely fixed onto a coverslip, and 10 µL of AHL was added. Using a Zeiss LSM700 microscope equipped with a 40× water immersion objective (W Plan-Apochromat 40×/1.0 DIC M27), an excitation laser (488 nm), and a detector for emissions that passed through a 555 nm short-pass filter, recordings of changes in GCaMP6 intensity were performed. During these recordings, the CS+ and CS– odors were delivered through a custom-made odor delivery device. The sequence involved delivering room air for 10 s, followed by an 8 s exposure to the CS+ odor. This was followed by another 10 s release of the room air followed by an 8 s presentation of the CS– odor. The recording was concluded with another 10 s delivery of the room air. Optical slices with a resolution of 512 × 512 pixels were continuously monitored for a period of 46 s at a rate of 2.17 frames per second. Changes in GCaMP6 fluorescent intensity in response to CS+ vs. CS− odors were quantified using the formula log10 (ΔF_CS+_/ΔF_CS−_). Intensity maps were generated for all functional calcium imaging studies using the Fiji (ImageJ) software.

### Quantitative reverse transcription polymerase chain reaction (qRT-PCR)

*Ldh* expression levels in the fly head and body were evaluated using qRT-PCR. Wild-type flies were collected and cultured in *Lactobacillus*-supplemented food for five days. RNA was extracted from the heads or bodies of adult flies using the TRIzol reagent (Invitrogen). SuperScript III First-Strand Synthesis SuperMix (Thermo Fisher Scientific) was used for first-strand cDNA synthesis from the extracted RNA. qRT-PCR was performed with Power SYBR Green PCR Master Mix (Thermo Fisher Scientific) using StepOnePlus Real-Time PCR System (Applied Biosystems) and StepOne software. Ribosomal protein L32 (RPL32) was used as the internal control. The following primers were used: LDH-fwd: 5’-AGATCCTGACTCCCACCGAA-3’, and LDH-rev: 5’-GCCTGGACATCGGACATGAT-3’.

### Statistical analysis

All statistical analyses were performed using the GraphPad Prism 9 software (GraphPad).

Comparisons between two different groups were performed using the paired Student’s *t*-test. Comparisons more than two groups were performed using one-way analysis of variance (ANOVA) and Tukey’s multiple comparison test. Statistical significance was defined as *p* < 0.05. All data were presented as the mean ± standard error of the mean (SEM).

## Supplementary Material

Supplemental Material

## Data Availability

The authors confirm that the data supporting the findings of this study are available within the article and its Supplementary Material. The datasets generated for this study can be found in the https://reurl.cc/G4ANrG
