# Supplementary material for: Probiotic Lactobacillus spp. improves Drosophila memory by increasing lactate dehydrogenase levels in the brain mushroom body neurons
Source: Gut Microbes. 2024 Feb 19;16(1):2316533. doi: 10.1080/19490976.2024.2316533 (PMC10877976; doi:10.1080/19490976.2024.2316533)
Supplement: Supplemental Material [file KGMI_A_2316533_SM5881.pdf]

Supplementary Material:

Title: Probiotic *Lactobacillus* spp. improves *Drosophila* memory by increasing lactate dehydrogenase levels in the brain mushroom body neurons

Shuk-Man Ho, Wan-Hua Tsai, Chih-Ho Lai, Meng-Hsuan Chiang, Wang-Po Lee, Hui-Yu Wu, Pei-Yi Bai, Tony Wu, Chia-Lin Wu

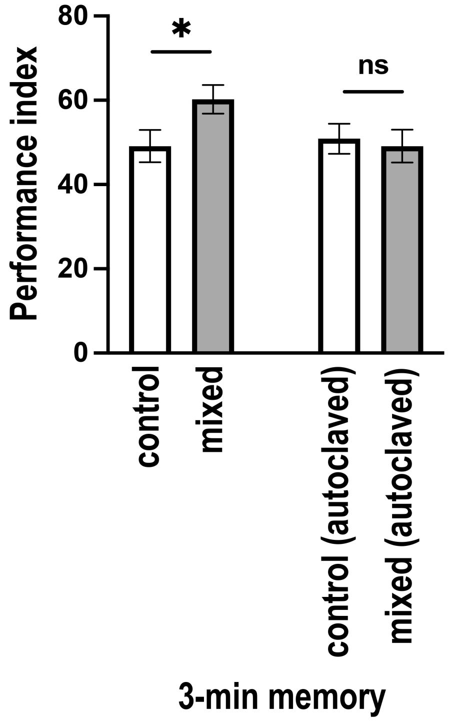

Figure S1. Flies did not show increased memory performance after feeding on heat-killed GMNL-185/GMNL-680.

The 3-minute memory performance of flies was evaluated under different conditions: unheated control, unheated GMNL-185/GMNL-680 mixed feeding, autoclaved (121 °C for 30 minutes) control, and autoclaved GMNL-185/GMNL-680 mixed feeding. Flies in the autoclaved GMNL-185/GMNL-680 mixed feeding group did not exhibit significant differences in memory performance compared to the control group. Each bar represents the mean ± SEM (N = 8 for each bar). *$p < 0.05$; non-significant difference (ns), $p > 0.05$, analyzed using the Student's *t*-test.

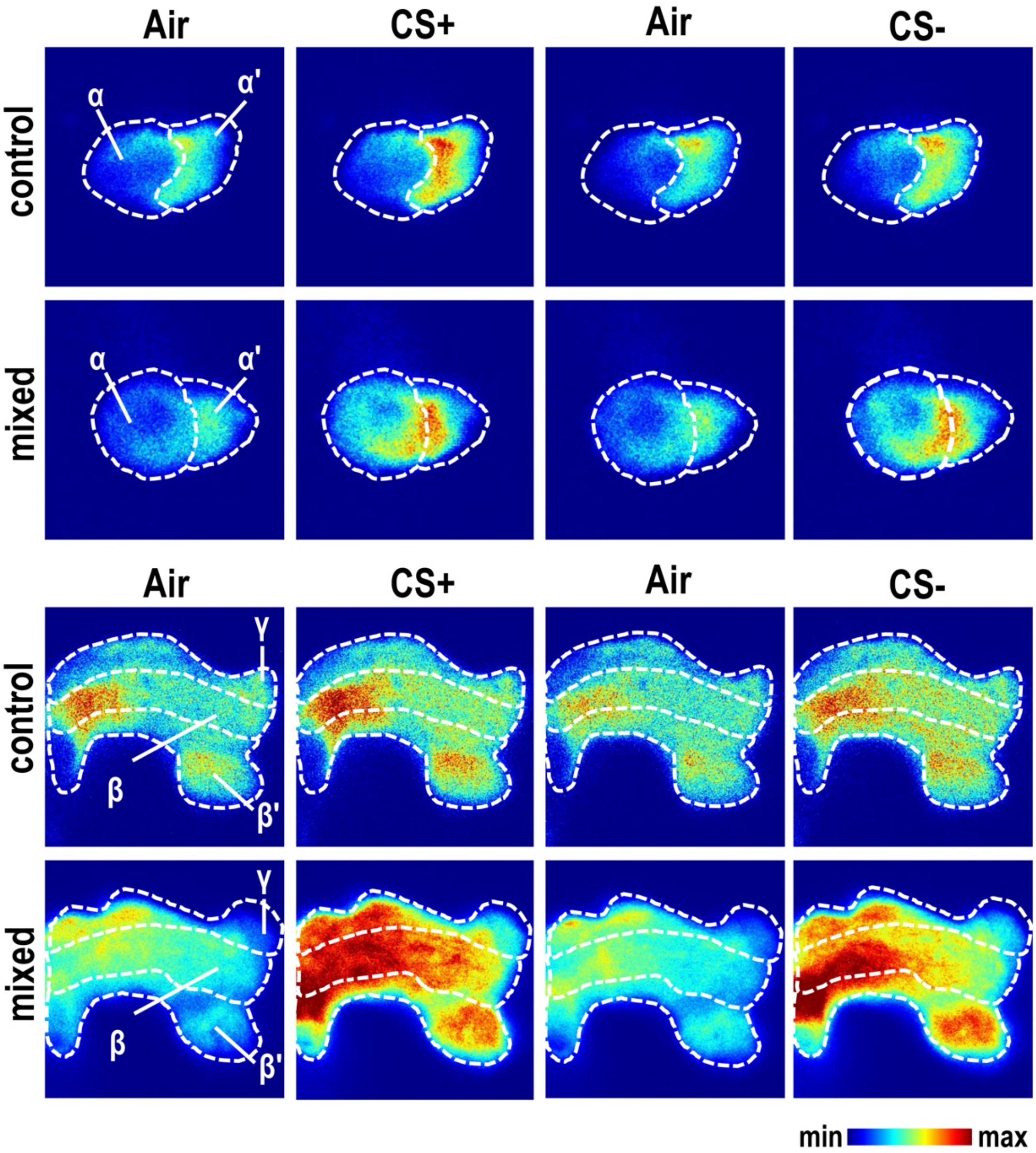

Figure S2. Pseudocolor images of odor responses in each mushroom body lobe. Flies carrying *R13F02-GAL4 > UAS-GCaMP6m* transgenes were fed with GMNL-185/GMNL-680 (labeled as "mixed") or with corn power (labeled as "control") for five days. Calcium responses to CS+ or CS- odor were analyzed at 1 hour after milder training conditions (three times 40-volt electrical shock plus CS+ odor for 1 minute). Recordings were made from optical sections in regions of the vertical (α' and α lobes) and horizontal (β', β, and γ lobes) of the mushroom body. The quantification of each data point is shown in Figure 4.

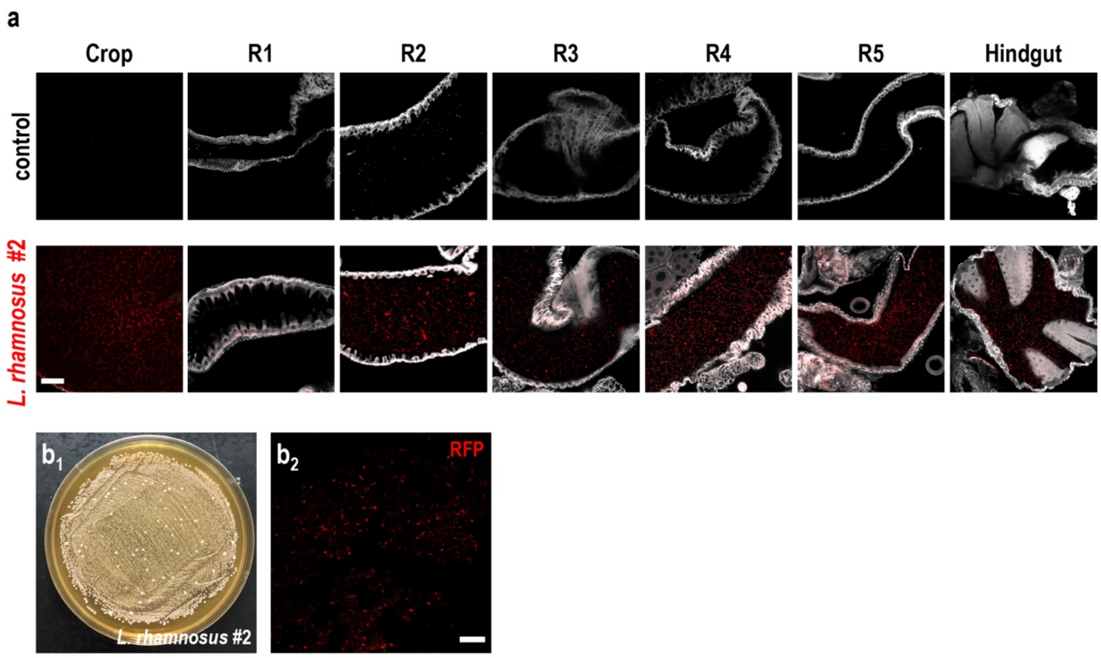

Figure S3. Live *L. rhamnosus* #2 in the fly digestive tracts after five days of probiotic feeding.

(a) RFP-tagged *L. rhamnosus* #2 was cultured in *Lactobacillus*-supplemented fly food medium. Wild-type flies were fed with a food medium containing RFP-tagged *L. rhamnosus* #2 for five days, and their digestive tracts were dissected and mounted on coverslips. RFP fluorescent signals were detected in the crop, R2, R3, R4, R5, and hindgut sub-regions of their digestive tracts. In contrast, the control group of flies were fed with regular food without *Lactobacillus* spp. for five days. No RFP signals were observed in the digestive tracts of the control group. The samples were immunostained with the anti-βPS-integrin antibody (grey). Scale bar = 50 μm. **(b)** After five days of feeding with a medium containing RFP-tagged *L. rhamnosus* #2, the fly digestive tracts were isolated and homogenized in PBS. The resulting supernatants were spread on MRS agar plates and incubated at 37°C for 2–3 days. Colonies of RFP-tagged *L. rhamnosus* #2 **(b_1)** were cultured on MRS agar plates. Individual colonies were isolated from the cultured MRS agar plates and placed on coverslips for confocal microscopy.

The cultured bacterial colonies expressed RFP fluorescent signals under confocal microscopy **(b_2)**. Scale bar = 20 μm.

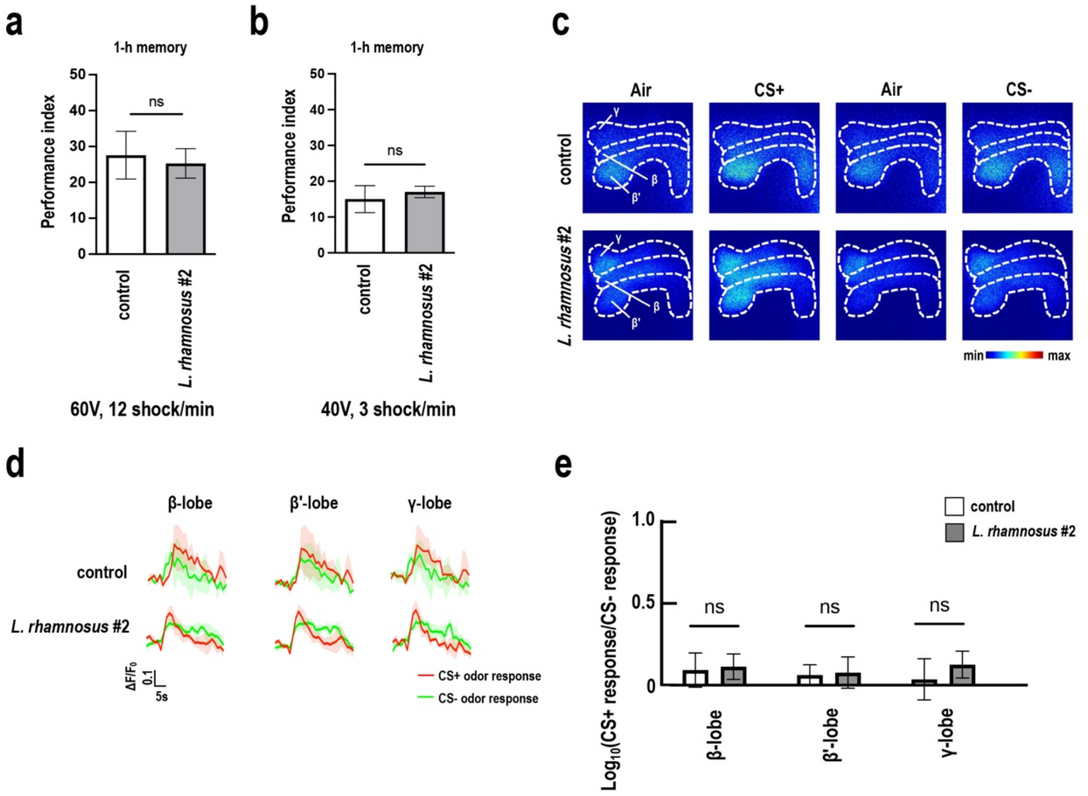

Figure S4. ***L. rhamnosus* #2 feeding did not affect olfactory memory and mushroom body neuronal response to training odor.**

In **(a)** regular training conditions (12 instances of 60-volt electrical shock with CS+ odor for 1 minute) or **(b)** milder training conditions (3 instances of 40-volt electrical shock with CS+ odor for 1 minute), flies did not show significant differences in performance index of 1-hour memory in *L. rhamnosus #2* feeding groups as compared to the control group. Each value represents the mean ± SEM (N = 6-7). Non-significant difference (ns), $p > 0.05$, determined using Student's *t*-test. **(c-d)** Flies carrying *R13F02-GAL4 > UAS-GCaMP6m* transgenes were fed with *L. rhamnosus* #2 or with corn power (labeled as "control") for five days. Calcium responses to CS+ or CS- odor were analyzed at 1 hour after milder training conditions. Recordings were made from optical sections in regions of the horizontal (β, β', and γ lobes) of the mushroom body. **(e)** Quantification of the GCaMP6 responses to the CS+ odor relative to the CS− odor at 1 h following a milder training protocol. The different regions of the horizontal of

the mushroom body lobes were analyzed, respectively. The logarithmic ratios of the CS+ response to the CS− response were computed using the peak response amplitudes. The *L. rhamnosus #2* feeding group flies did not alter calcium responses to the training odor (CS+) compared to the non-training odor (CS−) in the β, β' and γ lobes. There are no significant differences in logarithmic ratios of the CS+ response to the CS− response between the *L. rhamnosus #2* feeding group and non-feeding (control) group. Each value represents the mean ± SEM (N = 6). Non-significant difference (ns), $p > 0.05$, determined using Student's *t*-test.

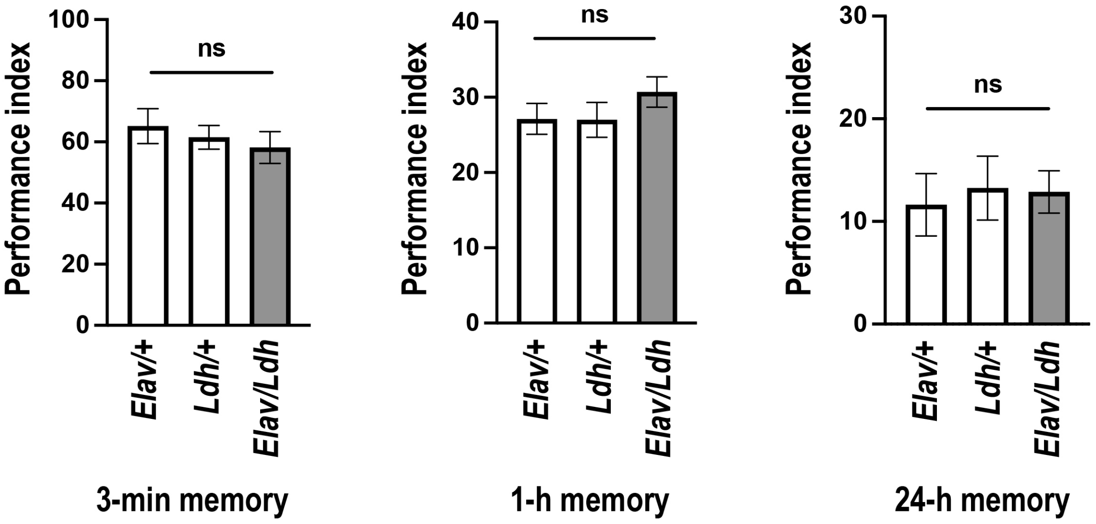

Figure S5. Pan-neuronal *Ldh* overexpression does not enhance olfactory memory. Overexpression of *Ldh* in neurons via *Elav-GAL4* did not significantly affect the 3-minute (N = 6), 1-hour (N = 8–10), and 24-hour (N = 8) memory performance compared to control groups. Each value represents the mean ± SEM. Non-significant differences (ns), $p > 0.05$, analyzed using the one-way ANOVA.

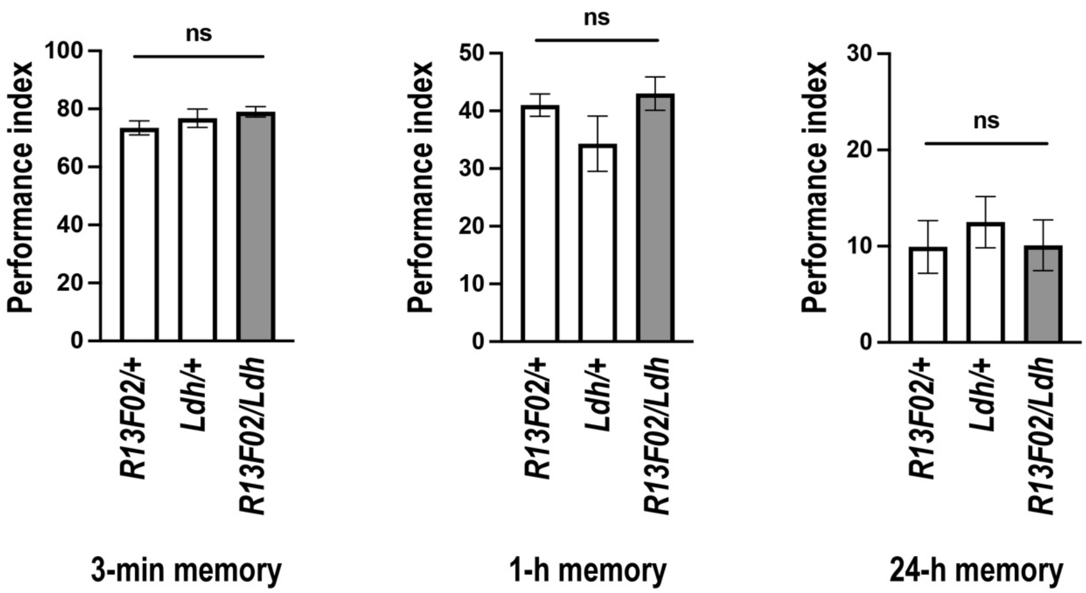

Figure S6. Mushroom body-specific *Ldh* overexpression does not enhance olfactory memories.

Overexpression of *Ldh* in the mushroom body neurons via *R13F02-GAL4* did not significantly affect the 3-minute (N = 9), 1-hour (N = 10), and 24-hour (N = 10) memory performance compared to the control groups. Each value represents the mean ± SEM. Non-significant differences (ns), $p > 0.05$, analyzed using one-way ANOVA.
